# Multigene signatures for early breast cancer in clinical practice: A report of the Lombardy genomic assays for breast cancer working group

**DOI:** 10.3389/fonc.2023.1081885

**Published:** 2023-03-06

**Authors:** Luca Licata, Deborah Cosentini, Rita De Sanctis, Monica Iorfida, Elena Rota Caremoli, Andrea Vingiani, Edda Lucia Simoncini, Giancarlo Pruneri, Elisabetta Munzone, Giampaolo Bianchini, Alberto Zambelli, Carlo Tondini

**Affiliations:** ^1^ Department of Medical Oncology, San Raffaele Hospital, Milan, Italy; ^2^ Faculty of Medicine and Surgery, Vita-Salute San Raffaele University, Milan, Italy; ^3^ Medical Oncology Unit, ASST Spedali Civili of Brescia, Brescia, Italy; ^4^ Medical Oncology and Hematology Unit, IRCCS - Humanitas Research Hospital, Milan, Italy; ^5^ Department of Biomedical Sciences, Humanitas University, Milan, Italy; ^6^ Division of Medical Senology, IEO, European Institute of Oncology, Milan, Italy; ^7^ Oncology Unit, ASST Papa Giovanni XXIII, Bergamo, Italy; ^8^ Department of Pathology and Laboratory Medicine, Fondazione IRCCS Istituto Nazionale dei Tumori, Milan, Italy; ^9^ Faculty of Medicine and Surgery, University of Milan, Milan, Italy; ^10^ SSVD Breast Unit, ASST Spedali Civili of Brescia, Brescia, Milan, Italy

**Keywords:** genomic test, ER+/HER2- breast cancer, early breast cancer, adjuvant therapy, multigene signatures

## Abstract

The increasing understanding of breast cancer biology has provided the basis for the development of multigene signatures aimed to improve the capability of clinicians to assess patients’ prognostication and risk stratification. Incorporating these tools in clinical practice has profoundly impacted on the decision-making process for the adjuvant therapy of patients with ER+/HER2- early breast cancer and the results from prospective adjuvant trials have strengthened the clinical utility of multigene signatures in this setting. In July 2019, Lombardy was the first Region in Italy to reimburse genomic testing for patients with ER+/HER2- early breast cancer. Three years later, a group of investigators from six referral Cancer Centers in Lombardy convened to debate the use of multigene signatures in clinical practice and share their own experience with the tests after reimbursement. Here, we reviewed relevant data on the role of multigene signatures in tailoring adjuvant chemotherapy for patients with ER+/HER2- early breast cancer and discussed about the optimal use of these assays in current clinical practice. As the treatment landscape of early breast cancer evolves and novel questions about the possible additional applications of multigene assays arise, we also provide our viewpoint on the potential implementation of the assays in the evolving scenario ER+/HER2- early breast cancer treatment.

## Introduction

The decision-making process on adjuvant treatment for patients with estrogen receptor positive and human epidermal growth factor receptor 2-negative (ER+/HER2-) early breast cancer may prove challenging. Clinicians should estimate the individual risk of recurrence and weigh the predicted benefit of adjuvant therapies against their short- and long-term toxicities in order to avoid both over- and undertreatment. The careful assessment of the classical clinicopathologic variables is essential to estimate the risk of disease recurrence but is little informative for prediction of chemotherapy benefit for individual patients (1).

The increasing understanding of breast cancer biology has led to the development of multigene signatures that provide prognostic information independent of that provided by standard clinicopathologic features and may help clinicians to better identify those patients with low-risk disease who can be safely spared the toxic effects of chemotherapy (2). All the commercially available signatures have been robustly clinically validated (3) and a plethora of studies consistently showed that their use can lead to a decrease of chemotherapy recommendation in up to 50% of cases (4–10). Accordingly, major Guidelines recommend the use of multigene signatures as a tool to tailor adjuvant chemotherapy decision (11, 12).

Based on this striking evidence, in July 2019 Lombardy was the first Region in Italy to reimburse genomic testing for patients with ER+/HER2- early breast cancer (13). The indication for regional reimbursement was for patients with formal recommendation to adjuvant chemotherapy after multidisciplinary discussion. In April 2022, a group of investigators from six referral Cancer Centers in Lombardy convened to debate the use of multigene signatures in clinical practice and shared their own experience with the tests after reimbursement. This latter point will be reported elsewhere. Here, we reviewed evidence from the literature about the four multigene assays available in Italy and discussed about their optimal use and potential implementation in the evolving field of adjuvant treatment of ER+/HER2- breast cancer.

## Which is the preferred test to tailor adjuvant treatment decision making?

Several multigene assays have been developed over the past two decades to guide adjuvant therapy decisions. Among them, Oncotype DX, MammaPrint, EndoPredict and Prosigna are the tests currently available in Italy (**Table 1**).

**Table 1:** Available Genomic Assays for ER+/HER2- Early Breast Cancer in Italy.

Genomic test	Description	Training set	Validation set	Results	Prospective validation	Ref
**Oncotype DX**	- 21-gene signature (RT-PCR) - Central laboratory - Low (RS 0-25) vs. high (RS 26-100) risk*	447 ER+/− tumor samples from pts with LN+/− disease enrolled in three clinical trials, including the NSABP B-20 tamoxifen only arm	668 ER+ tumor samples from pts with LN− disease in the tamoxifen only arm of NSABP B-14	10-year distant-recurrence: - 6.8% in low risk - 14.3% in intermediate-risk - 30.5% in high-risk	Yes (TAILORx, RxPONDER)	(14)
**MammaPrint**	- 70-gene signature (NGS-Illumina) - Central laboratory - Low (index 0.001 to 1.000) vs high (index −1.000 to 0) risk	78 ER+/− tumor samples <5 cm from pts <55 years of age with LN- disease	295 ER+/− tumor samples <5 cm from pts <53 years of age with and LN+/− disease (including samples from the training set)	Ten-year overall survival: - 54.6% in high-risk - 94.6% in low-risk	Yes (MINDACT)	(15)
**EndoPredict**	- 11-gene signature (RT-PCR) - Local laboratory - EPclin score: Low (score < 3.3) vs. high (‗ 3.3-6) risk	964 ER+ tumor samples from pts with LN+/− disease treated with tamoxifen	ER+ tumor samples from 378 pts with LN+/− disease from the ABCSG-6 trial (tamoxifen-only arm) and 1,324 pts from the ABCSG-8 trial	10-year distant recurrence: • ABCSG-6 - 4% in low-risk - 28% in high risk • ABCSG-8 - 4% in low-risk - 22% in high risk	No	(16)
**Prosigna**	- 50-gene signature (N-Counter) - Local laboratory - Low (0-40) vs. intermediate (41-60) vs. high (61-100) risk	189 ER+/− tumor samples from pts with LN+/− disease and 29 nonmalignant samples	ER+/− tumor samples from 761 pts with LN+/− disease who had not received adjuvant therapy and 133 pts who had received neoadjuvant chemotherapy	HR for RFS relative to luminal A: - 1.33–1.79 basal-like - 2.53–3.25 HER2-enriched - 2.43–2.88 luminal B Prediction of pCR: - 94% sensitivity - 43% PPV - 97% NPV	No	(17)

RT-PCR, Reverse Transcriptase-Polymerase Chain Reaction; ER, estrogen receptor; pts, patients; LN, lymph node; HR, hazard ratio; RFS, recurrence-free survival; pCR, pathological complete response; PPV, positive predictive value; NPV, negative predictive value.

*Oncotype DX Recurrence Score was initially categorized according to three risk groups: Low (RS 0-17), intermediate (RS 18-30) and high (RS 31-100). Subsequently, the TAILORx trial’s results redefined the cutoffs and eliminated the intermediate risk category, as shown in the table.

### Oncotype DX

**Figure 1:**
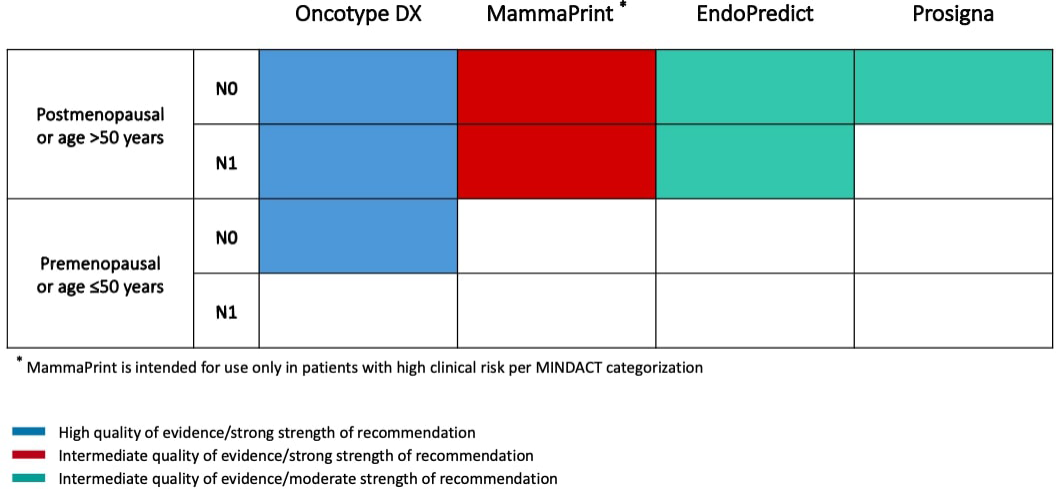
Summary of ASCO Guideline on the use of genomic assays to guide decisions on adjuvant therapy for patients with ER+/HER2- early breast cancer.

Oncotype DX (Exact Sciences; formerly Genomic Health) was among the earliest clinically validated genomic assay for use in patients with early breast cancer. This assay measures the expression of 16 tumor-associated genes and 5 control genes to compute a Recurrence Score (RS) from 0 to 100, resulting in assignment of patients into low-risk (RS <18), intermediate-risk (RS 18 to 30) or high-risk (RS ≥31) groups (**Table 1**) (14). In the initial validation study, the prognostic role of Oncotype DX was demonstrated using tumor samples from tamoxifen-treated patients in the NSABP B-14 trial, showing a significant difference in 10-year distant-recurrence rates between the three risk categories (14). Moreover, the RS has been shown to be also predictive of chemotherapy benefit in both node-negative and node-positive patients enrolled in the NSABP B-20 and SWOG-8814 trials, respectively (18, 19). The prognostic and predictive role of Oncotype DX has been prospectively validated in the TAILORx (20) and RxPONDER (21) trials, which enrolled 10,273 node-negative and 5,083 1-3 node-positive patients, respectively. Using more conservative cutoffs to minimize the potential for undertreatment (i.e. RS < 10 for low-, 11-25 for intermediate- and ≥ 26 for high-risk), these two trials showed that node-negative patients > 50 years and postmenopausal node-positive patients with RS 0-25 had no chemotherapy benefit (20, 21). In contrast, there was a detectable benefit from adjuvant chemotherapy in node-negative patients ≤ 50 years if the RS was 16-25 in TAILORx (20), and in premenopausal patients with 1-3 positive lymph nodes irrespective of RS in RxPONDER (21). According to these data, ASCO Guidelines suggest the use of Oncotype DX to guide decisions for adjuvant therapy in node-negative patients irrespective of age or menopausal status and in postmenopausal patients with 1-3 positive lymph nodes (**Figure 1**) (11).

### Mammaprint

MammaPrint (Agendia) is a microarray-based 70-gene assay performed by a central laboratory in Amsterdam. This assay stratifies patients into low-risk or high-risk prognostic groups and was first validated in 295 breast cancer patients both ER-positive and ER-negative (**Table 1**) (15). After extensive clinical validation in many other studies involving both node-negative and node-positive patients, the clinical utility of MammaPrint was evaluated in the prospective MINDACT trial, which enrolled 6,693 patients across nine European countries between 2007 and 2011 (22). Patients with discordant genomic and clinical risk were randomly assigned to endocrine therapy or chemo-endocrine therapy. The trial met its primary objective, since the lower boundary of 95% CI for the 5-year distant metastasis-free survival (DMFS) in patients with clinically high risk and genomic low risk tumors who did not receive adjuvant chemotherapy was above the prespecified 92% (5-year DMFS 94.7% [95% CI, 92.5-96.2]), and there was a not significant 1.5% difference in the 5-year DMFS between patients who received or not received chemotherapy (22). An update of the trial reported results according to age and showed that adjuvant chemotherapy was associated with a significant 5.0% absolute benefit in 8-year DMFS in patients ≤ 50 years, while no benefit was observed in older patients (23). On the basis of these data, ASCO Guidelines do not recommend the use of MammaPrint in patients ≤ 50 years (**Figure 1**) (11). It is noteworthy that patients with low clinical risk and high genomic risk enrolled in MINDACT did not derive a benefit from the addition of adjuvant chemotherapy to endocrine therapy, questioning the utility of the test as a predictive biomarker for chemotherapy benefit.

### EndoPredict

EndoPredict (Myriad Genetics) is an assay marketed in Europe as a diagnostic kit that can be performed by local laboratories. The test measures the expression of 8 cancer-related and 3 reference genes using RT-PCR, in order to calculate a risk score and stratify patients into a low- or high-risk of recurrence group, if treated with adjuvant endocrine therapy alone (**Table 1**). The resulting score has also been combined with tumor size and nodal status to derive a comprehensive risk score, the EPclin, that has been validated in more than 1,700 patients from the randomized ABCSG-6 and ABCSG-8 phase III trials (16). In a nonrandomized analysis of patients treated with adjuvant endocrine therapy compared with those treated with chemo-endocrine therapy, high-risk EPclin has been shown to predict a significant benefit from the addition of chemotherapy to endocrine therapy (10-year DRFI 12% vs 20%). However, data from randomized, prospective trials establishing the predictive value of the assay are still lacking. Furthermore, EPclin has also been shown to identify patients who are at risk for late recurrence as well as those with a favorable long-term prognosis after adjuvant endocrine therapy (24). Nevertheless, ASCO Guidelines do not recommend the test to guide decisions about extended endocrine therapy (11).

### PAM50

PAM50 (Prosigna) is a diagnostic kit that uses Nanostring technology to quantify mRNA expression of 50 genes used in the molecular subtypes’ classification of breast cancer. When integrated with a proliferation score and tumor size, the test leads to the risk-of-recurrence (ROR) score, that reflects the 10-year risk of distant recurrence **(** **Table 1** **)** (17). The prognostic value of Prosigna has been demonstrated in several retrospective analyses of prospective studies, but currently there are no data from prospective randomized trials that established its clinical utility.

The ongoing OPTIMA trial (ISRCTN42400492), which randomized patients with ER+/HER2- early breast cancer (pN1-2 or pN1mi with pT ≥20mm or pN0 with pT ≥30mm) to receive chemo-endocrine therapy or to undergo Prosigna testing and omit chemotherapy in case of low- or intermediate-Prosigna Score (≤ 60), hopefully will provide information about the clinical utility of this assay as a predictive biomarker.

We agreed that all signatures are valid prognostic tools useful to identify low-risk patients with good outcome with adjuvant endocrine therapy alone. However, according to the abovementioned data, only Oncotype DX has been shown to provide convincing predictive information for chemotherapy benefit in prospective trials and is the only one recommended for both post- and premenopausal patients with a level of evidence and a grade of recommendation of I, A (11).

For these reasons, Oncotype DX resulted the preferred test chosen by the Panelists in almost all cases (1098 of 1132 patients who received an indication for genomic testing in the last two years).

## Could the workflow of test prescription be improved?

Modern breast cancer management has become increasingly complex and requires dedicated breast cancer specialists working together to deliver high-quality care throughout the patient journey. The multidisciplinary team (MDT) -defined by the UK Department of Health as a ‘‘group of people of different health care disciplines, which meets together at a given time to discuss a given patient and who are each able to contribute independently to the diagnostic and treatment decisions about the patient’’ (25)- is fundamental in breast cancer management and represents a benchmark for accreditation and funding (26). Different benefits have been associated to MDT-based management: higher patient satisfaction, closer adherence to evidence-based guidelines and, more importantly, better patients’ outcome (27–29). In the context of genomic assays, the fundamental role of the MDT is to identify patients eligible for the test. Accordingly, the prescription of the test usually occurs after MDT discussion of a given patient who underwent surgery and whose histological report is available at the time of the meeting.

However, this process sometimes might lead to different indications for genomic testing by different MDTs on one side, and to a delay in the initiation of the adjuvant systemic therapy beyond the 3-6 weeks recommended by Guidelines (12) one the other side. This latter point might be of particular relevance when MDT decides to order a test to be performed in a central lab.

In this view, we advocated alternative paths for genomic test prescription in an effort to standardize practice and reduce delays to adjuvant chemotherapy initiation. We endorsed the “Oncotype DX reflex testing”, that is the automated preparation and shipping of tumor sample by the pathologist at the time of the histological examination (therefore, earlier the MDT discussion) in cases that meet pre-defined criteria. The implementation of Oncotype DX reflex testing in clinical practice has been shown to improve the timeliness to chemotherapy initiation (30), with potential positive implications in patient satisfaction and outcome.

We endorse the reflex testing for postmenopausal patients < 70 years with ER+/HER2- breast cancers, both node-negative and 1-3 node positive and the following tumor characteristics: T1c-2, Grade 2-3 and at least one of Ki67 21-40% or PgR < 20%. We acknowledge that advanced age, by itself, should not be a criterion to preclude a patient from receiving the most appropriate anticancer treatment and that ‘functional’ rather than chronological age should be considered in the decision making about adjuvant therapy. However, older patients require a more careful evaluation of the risk-benefit ratio of any medical intervention, including a geriatric assessment and a careful consideration of life expectancy and competing risks of mortality that should be performed by the various members of the MDT (31).

## Could Ki67 analysis avoid the use of genomic testing?

No, it doesn’t. Ki67 is a quantitative measure of proliferation and, along with routine IHC measurements of ER, PgR and grade, is useful to classify ER+/HER2- breast cancers with the surrogate definitions of ‘luminal A-like’ (strong ER/PR expression, lower Ki67, lower grade) or ‘luminal B-like’ (lower levels of ER/PR expression, higher Ki67, higher grade) (32). However, while hormone receptor and tumor grade assessment are largely standardized, there is persistent controversy on the optimal thresholds to define “low” and “high” Ki67 values. According to the International Ki67 in Breast Cancer Working Group recommendations, a full agreement on defining a case as “low” or “high” Ki67 exists only for values of 5% or less or 30% or greater, respectively, and these thresholds could be used to withhold or proceed with chemotherapy (33). However, since most ER+/HER2- tumors fall between these two extremes (34), these cutoffs are often of limited clinical applicability. In our and other experience (10, 35), a substantial proportion of tumors with Ki67 > 30% has a low RS and therefore a recommendation of adjuvant chemotherapy for these patients based solely on high Ki67 values would have been questionable. Recent analyses from the TransATAC study showed that Oncotype DX is substantially an estrogen-driven signature, since the correlation between RS and its proliferation module is much weaker than the strong negative correlation between RS and its estrogen module (36). Therefore, it should not surprise if high RS values have been found also in tumors with low Ki67 levels and this has led to a change in recommendation from endocrine therapy to chemo-endocrine therapy (10, 37). Indeed, convincing evidence of the predictive value of Ki67 for chemotherapy benefit is still lacking (38). Accordingly, ASCO Guidelines recommend that Ki67 should be used only as a prognostic biomarker to define individual risk of recurrence (11) and a large fraction of the 2021 St Gallen Panelist believes that a Ki67 threshold for recommending chemotherapy in ER+/HER2- breast cancer is simply not known (32). We acknowledge that Ki67 assessment is an easy and inexpensive method to evaluate tumor cell proliferation that remains extremely useful in many regions of the world with limited access to genomic assays. Nevertheless, we suggest that, if available, genomic assays be employed in those tumors where uncertainty on the benefit from adjuvant chemotherapy still remains despite Ki67 levels.

## Which is the role of genomic testing in premenopausal patients?

Age at diagnosis is an important prognostic factor in ER+/HER2- early breast cancer (39) and studies suggested that, among the luminal subtype, tumors arising in young women may be a biologically distinct disease (40). Nevertheless, the role of age and menopausal status has been quite neglected in the development of multigene signatures and the data from validation studies should therefore be interpreted keeping in mind this caveat.

Concerning Oncotype DX, both TAILORx and RxPONDER have found significant interaction for outcome between chemotherapy effect and age or menopausal status (20, 21). In TAILORx, subgroup analysis of women ≤ 50 years old with an RS of 16–25 showed lower rates of distant recurrence with chemo-endocrine therapy compared with endocrine therapy alone. Specifically, women with an RS of 16-20 and high clinical risk (as per MINDACT categorization) and women with an RS of 21-25 irrespective of clinical risk had an absolute reduction in the rate of distant recurrence of approximately 6.5% at 9 years associated with adjuvant chemotherapy (20, 41). Interestingly, this chemotherapy benefit in young women was almost confined to premenopausal women between 46 and 50 years old and waned at younger and older ages and with menopause, suggesting that the benefit is likely due to chemotherapy-induced ovarian function suppression (41). RxPONDER results further corroborate this hypothesis. In that trial, premenopausal women with 1-3 positive lymph-nodes and an RS of 0-25 had a statistically significant and clinically meaningful 40% risk reduction of an IDFS event with chemo-endocrine therapy compared to endocrine therapy alone (5-year IDFS 93.9% vs 89.0%; HR 0.60; 95% CI, 0.43 to 0.83; P=0.002), while postmenopausal women derived no benefit from chemotherapy (5-year IDFS 91.3% vs 91.9%; HR 1.02; 95% CI, 0.82 to 1.26; P=0.89) (21). Somehow consistently with the hypothesis that the benefit of chemotherapy might be primarily driven by its endocrine effect of ovarian function suppression, in premenopausal women who were 50 years of age or older (women who are likely to reach menopause early, with or without chemotherapy), no benefit was observed (21). Similarly to TAILORx and RxPONDER, the updated results of the MINDACT trial showed that patients ≤ 50 years of age derived a significant 5.0% benefit in distant metastasis-free survival (DMFS) with chemo-endocrine therapy compared to endocrine therapy alone (8-year DMFS 93.6% vs 88.6%; HR 0.54; 95% CI, 0.30 to 0.98), while no benefit was observed in older patients (8-year DMFS 90.2% vs 90.0%; HR 0.82; 95% CI, 0.55 to 1.24) (23). It is noteworthy that the chemotherapy benefit in MINDACT was not observed at an earlier follow-up (22), and this indirectly suggests that the benefit is unlikely to be due to a greater cytotoxic effect of chemotherapy in young women, which is known to occur in the first few years after administration (42). Unfortunately, none of these trials was designed to answer the question whether chemotherapy can be replaced by ovarian function suppression in premenopausal women. In all trials, the rate of ovarian function suppression in the endocrine therapy alone arms was limited, and the treatment-induced ovarian suppression in the chemotherapy arms has generated a sort of imbalance in the type of endocrine modulation between arms that hampers the full applicability of the results in current clinical practice, where ovarian function suppression is often planned as a part of endocrine therapy. A definitive answer to this question would require a large adjuvant trial (such as the planned NRG Oncology’s BR009) fully dedicated to premenopausal patients with low genomic risk tumors and investigating whether chemotherapy added to an optimal endocrine therapy provides any benefit. Despite one might speculate that the biological peculiarities of tumors arising in young women (40) could determine a greater cytotoxic effect of chemotherapy, data from TAILORx (20, 41), RxPONDER (21) and MINDACT (23), together with other evidence from prospective trials (43, 44), support the hypothesis that most of the benefit observed with chemotherapy in young women might be due to its endocrine effect of ovarian suppression.

Although Guidelines recommend that genomic testing in premenopausal women with 1-3 positive lymph-nodes should not be offered to guide decisions for adjuvant therapy (11) and some clinicians have interpreted this recommendation as an indication for chemotherapy in all cases, we believe that Oncotype DX may be informative also in these cases and that low genomic risk patients may be considered for either chemotherapy or ovarian suppression based on a comprehensive risk assessment that includes also RS values. Nevertheless, young patients should be informed about the uncertainties in interpreting genomic test results and actively involved in the decision-making process on their optimal adjuvant therapy.

## Open questions and future directions of multigene assays

We reviewed relevant data about the role of multigene assays in tailoring the decision-making for adjuvant chemotherapy for patients with ER+/HER2- early breast cancer and provided our viewpoint on specific topics regarding the optimal use of these assays in clinical practice. Nevertheless, the treatment landscape of ER+/HER2- early breast cancer is evolving and novel questions about the potential additional applications of multigene assays are emerging.

### Firstly, could genomic tests have a role in neoadjuvant therapy decision-making?

Neoadjuvant therapy is increasingly used in the management of early breast cancer and the feasibility of performing gene expression profiling using tumor tissue from core biopsies has been largely demonstrated (45–48). Several studies (reviewed by Griguolo et al. (49)) investigated the role of multigene signatures in the neoadjuvant setting in recent years and, in this context, Oncotype DX was the most extensively studied assays. Collectively, these studies reported a consistent association between higher genomic risk and higher response to neoadjuvant chemotherapy and, symmetrically, between lower genomic risk and higher response to neoadjuvant endocrine therapy (49). However, since most of this evidence comes from small and/or retrospective studies, ASCO Guidelines recommend against the use of multigene signatures to guide neoadjuvant therapy decision-making (50). In contrast, St Gallen Panelists endorsed the use of genomic assays on core biopsies to aid in choosing between neoadjuvant chemotherapy or neoadjuvant endocrine therapy (32). Despite we acknowledge that genomic signatures should be applied very carefully in a context where axillary nodal involvement might be unclear, we endorse the idea that, in selected cases, these tools may be used also in the neoadjuvant setting.

### Second, could genomic tests be employed to inform endocrine therapy decision?

Probably yes. As noted above, while other signatures are dominated by proliferative features, Oncotype DX RS has a strong inverse correlation with its estrogen module (36) and this might explain the observation that patients with low RS have a greater reduction of disease recurrence with adjuvant tamoxifen and a higher clinical response rates to different types of neoadjuvant endocrine therapies (51, 52). Given the lower relative benefit from endocrine therapy in patients with higher RS, these patients might theoretically be eligible to endocrine treatment escalation strategies. This might be especially relevant for premenopausal patients, for whom incremental benefit with the addition of ovarian suppression to tamoxifen and with the substitution of tamoxifen with exemestane together with ovarian suppression has already been demonstrated in the SOFT and TEXT trials (53). The elegant Subpopulation Treatment Effect Pattern Plot (STEPP) analysis of these trials provides valuable insights to estimate the absolute benefit from the three endocrine therapy modalities according to baseline absolute risk, and the composite risk score derived by the investigators of SOFT and TEXT represents a useful tool for clinicians to tailor endocrine therapy decision-making for premenopausal patients (54). However, this tool does not take into account the potential differences in endocrine responsiveness that might derive from different biological features not captured by the composite risk. Although we recognize that Oncotype DX has not been developed to select the most appropriate adjuvant endocrine treatment, we believe that the role of the assay in this field may deserve further investigation.

### Finally, which is the role of genomic tests in the context of modern adjuvant therapies?

One attempt of treatment escalation in ER+/HER2- early breast cancer has been to combine adjuvant endocrine therapy with CDK4/6 inhibitors. The role of these agents in the early setting has been investigated in 4 large clinical trials (55–58). While the results from NATALEE (55) investigating ribociclib are pending, PALLAS and Penelope-B, the two trial investigating palbociclib, were both negative (56, 57). In contrast, in monarchE trial the addition of abemaciclib to endocrine therapy for patients with ER+/HER2- high-risk early breast cancer resulted in a significant 30% reduction in the risk of developing an IDFS event (58). Abemaciclib benefit was generally consistent across prespecified subgroups, including those stratified according to Ki67 levels (< 20% vs > 20%) (58), but data regarding genomic risk of patients are not available. Moreover, because of their high-risk clinicopathologic features, more than 95% of patients in monarchE has received prior neo-/adjuvant chemotherapy, so that the trial has fundamentally evaluated the efficacy of the addition of abemaciclib to chemo-endocrine therapy. Whether genomic signatures might aid to identify patients with differential relative benefit from CDK4/6 inhibitors or guide selection between CDK4/6 inhibitors and chemotherapy is an open and intriguing research question. The ongoing WSG-ADAPTcycle (NCT04055493), evaluating ribociclib plus endocrine therapy vs chemo-endocrine therapy in patients identified as intermediate risk (based on Oncotype DX RS and response to 3 weeks of preoperative endocrine therapy) who are not candidate to endocrine therapy alone, will contribute to get a clearer picture of this complex upcoming scenario. The investigators of the WSG-ADAPTcycle should be commended also for the inclusion of a subgroup of patients with N2-3 tumors, a population in which the role of genomic testing is currently unknown.

## Conclusion

The increasing understanding of breast cancer biology has provided the basis for the development of multigene assays aimed to improve our capability of patients’ prognostication and risk stratification. Incorporating these tools in clinical practice has profoundly changed the decision-making for adjuvant chemotherapy for patients with ER+/HER2- early breast cancer and the results from large prospective adjuvant trials have strengthened the clinical utility of multigene assays in this setting. As the treatment landscape of early breast cancer broadens, novel questions arise and new areas of research emerge. In this evolving scenario, the contribution of multigene assays to personalized medicine may become even more important.

## Data availability statement

The original contributions presented in the study are included in the article/supplementary material. Further inquiries can be directed to the corresponding author.

## Author contributions

All authors listed have made a substantial, direct, and intellectual contribution to the work and approved it for publication.
